# Discovery of disrupted sustained attention and altered functional connectivity in far‐from‐onset Huntington's disease gene‐expanded young adults

**DOI:** 10.1002/alz.70944

**Published:** 2026-01-13

**Authors:** Christelle Langley, Michela Leocadi, Nicola Z. Hobbs, Mena Farag, Michael J. Murphy, Kate Fayer, Rachael I. Scahill, James B. Rowe, Trevor W. Robbins, Sarah J. Tabrizi, Barbara J. Sahakian

**Affiliations:** ^1^ Department of Psychiatry University of Cambridge Cambridge UK; ^2^ Behavioural and Clinical Neuroscience Institute University of Cambridge Cambridge UK; ^3^ Huntington's Disease Centre, Department of Neurodegenerative Disease UCL Queen Square Institute of Neurology University College London London UK; ^4^ Department of Clinical Neurosciences University of Cambridge Cambridge UK; ^5^ Cambridge University Hospitals NHS Foundation Trust Cambridge UK; ^6^ Medical Research Council Cognition and Brain Sciences Unit University of Cambridge Cambridge UK; ^7^ Department of Psychology University of Cambridge Cambridge UK

**Keywords:** Huntington's disease, sustained attention, resting‐state fMRI

## Abstract

**BACKGROUND:**

Cognitive impairments are a hallmark of Huntington's disease (HD).

**METHODS:**

Seventy‐one participants (43 HD gene‐expanded [HDGE], 28 healthy controls) from the HD‐Young Adult Study at two timepoints ≈ 4.7 years apart, completed the Cambridge Neuropsychological Test Automated Battery Rapid Visual Information Processing task and underwent resting‐state functional magnetic resonance imaging. We focused on predefined regions of interest that are involved in sustained attention.

**RESULTS:**

HDGE individuals showed significantly poorer sustained attention than controls (*p_adj_* = 0.007), with no significant change over time. Functional connectivity (FC) analyses revealed group differences in attention‐related networks, including the occipital–operculum and lentiform–orbitalis pathways. Time and group × time effects were also observed in frontal and parietal regions.

**DISCUSSION:**

These findings demonstrate early and persistent attention deficits in HDGE, linked to altered FC in attention‐related circuits. This supports the presence of early cognitive dysfunction in HD and highlights potential compensatory and pathological changes in brain networks prior to the onset of clinical motor symptoms.

**Highlights:**

- We detail the discovery of early sustained attention deficits in Huntington's disease (HD) gene‐expanded (HDGE) young adults.
- These sustained attention deficits do not measurably decline over a 4.7‐year period.
- Altered functional connectivity was observed in attention‐related brain networks.
- Alterations in regions include occipital, opercular, lentiform, and frontal areas.
- Findings support attention as an early cognitive biomarker in HDGE young adults.

## 1 INTRODUCTION

Huntington's disease (HD) is a rare inherited neurodegenerative disorder characterized by movement, cognitive, and psychiatric symptoms. It is caused by an expansion of the cytosine–adenine–guanine (CAG) trinucleotide repeat in exon 1 of the huntingtin gene (*HTT*), resulting in the production of a mutant huntingtin protein.^1^ The larger the number of CAG repeats, the earlier the onset of HD symptoms.^2^ Diagnosis typically relies on the presence of significant motor abnormalities. However, models based on age and CAG repeat length can assist in the prediction of the onset of motor symptoms,^2^ allowing the study of individuals decades before predicted onset. A new classification system for the stages of HD has been developed, with stages 0, 1, and 2 representing HD gene expanded (HDGE) individuals.^3^ Individuals can exhibit cognitive, psychiatric, and brain changes detectable up to 15 years before clinical motor diagnosis,^4^, ^5^ and we have recently shown this at even earlier stages of the disease.^6^, ^7^ The neurodegeneration in presymptomatic HDGE individuals is well established and is particularly severe in the striatum,^4^, ^8^, ^9^ with loss of GABAergic medium spiny projection neurons.^10^ However, as the disease progresses into more advanced stages, neurodegeneration becomes more widespread in the cortex and white matter.^9^, ^11^

Given the early disruption of the frontostriatal networks in HD,^11^ much of the research into cognitive deficits has focused on the executive function of cognitive flexibility, which is subserved by these networks^12^, ^13^ and shows early disruption in HD.^14^, ^15^, ^16^ However, we have discovered that there are also early deficits in sustained attention, as measured by the Cambridge Neuropsychological Test Automated Battery (CANTAB) Rapid Visual Information Processing (RVP) task. Our previous study using the HD Young Adult Study (HD‐YAS) cohort, which is the earliest and furthest from motor‐onset adult HD group studied to date, detected an early subtle deficit in sustained attention in HDGE individuals compared to healthy controls (HCs). However, these tests did not survive correction for multiple comparisons in a broad neuropsychological battery.^6^ In our recent follow‐up study, 4.5 years later, this deficit in sustained attention had become more pronounced at the cross‐sectional level and survived multiple corrections.^7^ While attentional impairments have been documented in later stages of HD,^17^, ^18^ sustained attention deficits have not been extensively studied in the earliest stages of the disease. Previous research has predominantly focused on cognitive flexibility and other executive functions, with sustained attention often overlooked or assumed to remain intact until more advanced disease stages.

While the neural substrates of cognitive inflexibility in HD have been explored, the neural mechanisms underlying early sustained attentional impairments in HD remain poorly understood. Previous studies have shown that sustained attention performance in healthy individuals is predominantly supported by frontoparietal networks,^19^, ^20^ but also involves basal ganglia structures, including the lentiform nucleus,^20^ of which the putamen is part. Given that sustained attention is supported by regions known to be disrupted early in HD, it is critical to investigate whether and how these networks contribute to attentional deficits well before clinical motor diagnosis. Understanding these mechanisms could provide novel insights into the cognitive pathophysiology of HD and identify potential early cognitive biomarkers for intervention.

In the present study, we examined performance on the CANTAB RVP in a group of far‐from‐clinical‐motor‐onset HDGE participants in the HD‐YAS.^6^, ^7^ Specifically, we used resting‐state functional magnetic resonance imaging (fMRI) to examine the association between the functional connectivity (FC) in predefined frontoparietal networks and separate performance on the sustained attention detection threshold (RVP A') of the CANTAB RVP, both cross‐sectionally and longitudinally over a 4.5 year follow‐up. Based on our previous findings, we hypothesized that the HDGE group would show a deficit in sustained attention cross‐sectionally compared to HC but would not show longitudinal decline.^7^ In addition, the FC associated with RVP performance would be altered in the HDGE group compared to HCs.

## 2 MATERIALS AND METHODS

### 2.1 Participants

This study used a subset of right‐handed participants drawn from a larger cohort of 131 individuals (64 HDGE and 67 HCs) originally recruited as part of the HD‐YAS study,^6^ with 103 (57 HDGE and 46 HCs) returning for longitudinal follow‐up ≈ 4.5 years later.^7^ From this original cohort, all right‐handed participants with longitudinal resting‐state fMRI data were included. This resulted in a final sample of 71 (43 HDGE and 28 HCs; see Table 1). Participants were excluded if they were left‐handed (*n* = 10), lacked longitudinal imaging data (*n* = 16), or failed quality control (*n* = 6; Table S1 in supporting information for comparisons). Participants in the two groups were closely matched for age, sex, education, and IQ (measured by the National Adult Reading Test [NART]), and assessed by an experienced HD clinician at the National Hospital for Neurology and Neurosurgery, London, UK. HDGE participants showed no clinical motor signs of disease (Unified Huntington's Disease Rating Scale Total Motor Score [UHDRS TMS] ≤ 5) and had a disease burden score ≤ 240 (calculated as age × [CAG − 35.5]), corresponding to an estimated > 18 years from predicted clinical motor diagnosis. All HDGE participants were stages 0 or 1 on the HD Integrated Staging System (HD‐ISS; Figure S1 in supporting information).^3^ CAG repeat length was determined in a single laboratory. Controls were either expansion‐negative family members or individuals with no familial risk of HD. All participants provided written informed consent according to the Declaration of Helsinki, and the study was approved by the Bloomsbury Research Ethics Committee (reference 22/LO/0058).

**RESEARCH IN CONTEXT**

1. **Systematic review**: While early cognitive changes in Huntington's disease (HD) are well documented, no studies have longitudinally examined sustained attention or its neural correlates using resting‐state functional magnetic resonance imaging in HD gene‐expanded (HDGE) young adults. We discovered sustained attention deficits with a large effect size, which did not decline further over ≈ 4.7 years. We also determined the underlying neural basis of these deficits.
2. **Interpretation**: We demonstrate that sustained attention deficits in HDGE individuals are present early and decades prior to predicted clinical motor onset. These deficits are associated with altered functional connectivity (FC) in attention‐related brain networks, including occipital, opercular, and lentiform regions. Our findings suggest that deficits in sustained attention are robust and may possibly be evident prior to impairments in cognitive flexibility and are underpinned by distinct neural changes.
3. **Future directions**: Further research should explore whether these FC alterations can serve as early biomarkers and whether they represent neurodegenerative or neurodevelopmental processes.

**TABLE 1:** Demographics.

	HDGE (*n* = 43)	HC (*n* = 28)	*t* value/x2	*p* value	Effect size
Age (time 1)	30.13 (5.67)	29.81 (5.95)	−0.22	0.82	0.06
Age (time 2)	34.80 (5.61)	34.62 (6.11)	−0.12	0.90	0.03
IQ (NART)	102.86 (6.63)	105.93 (8.38)	1.63	0.11	0.42
Education (time 1)	4.23 (0.95)	4.43 (0.92)	0.87	0.39	0.21
Education (time 2)	4.67 (0.64)	4.71 (0.85)	0.21	0.83	0.05
Sex	48.84% Females (21)	57.14% Females (16)	0.20	0.66	0.00
Interval (years)	4.70 (0.54)	4.84 (0.63)	0.97	0.34	0.24
CAG	42.05 (1.54) 39‐46				
CAP100 (time 1)	55.72 (8.26) 41.57–76.90				
CAP100 (time 2)	64.55 (8.25) 48.55–86.70				
HD‐ISS stage 0 (time 1)	86.05% (37)				
HD‐ISS stage 1 (time 1)	13.95% (6)				
HD‐ISS stage 0 (time 2)	65.12% (28)				
HD‐ISS stage 1 (time 2)	34.88% (15)				

*Notes*: Age represented as mean (standard deviation); IQ NART represented as mean (standard deviation); Education—International Standard Classification of Education represented as mean (standard deviation); Sex—percentage (and count) of female participants in each group; Interval (years)—average time interval between time 1 and time 2 cognitive assessments (in years) represented as mean (standard deviation); CAG—Number of CAG repeats represented as mean (standard deviation) minimum‐maximum; CAP100—CAG‐Age Product scaled to 100 an index combining CAG repeat length and age to estimate disease burden represented as mean (standard deviation) minimum‐maximum; HD‐ISS stage represented as percentage (and count); time 1/time 2 indicate the two assessment points in the longitudinal study.

Abbreviations; CAG, cytosine–adenine–guanine; HC, healthy control; HDGE, Huntington's disease gene expansion; HD‐ISS, Huntington's Disease Integrated Staging System; IQ (NART), Intelligence Quotient assessed using the National Adult Reading Test.

### 2.2 CANTAB RVP task

**FIGURE 1:**
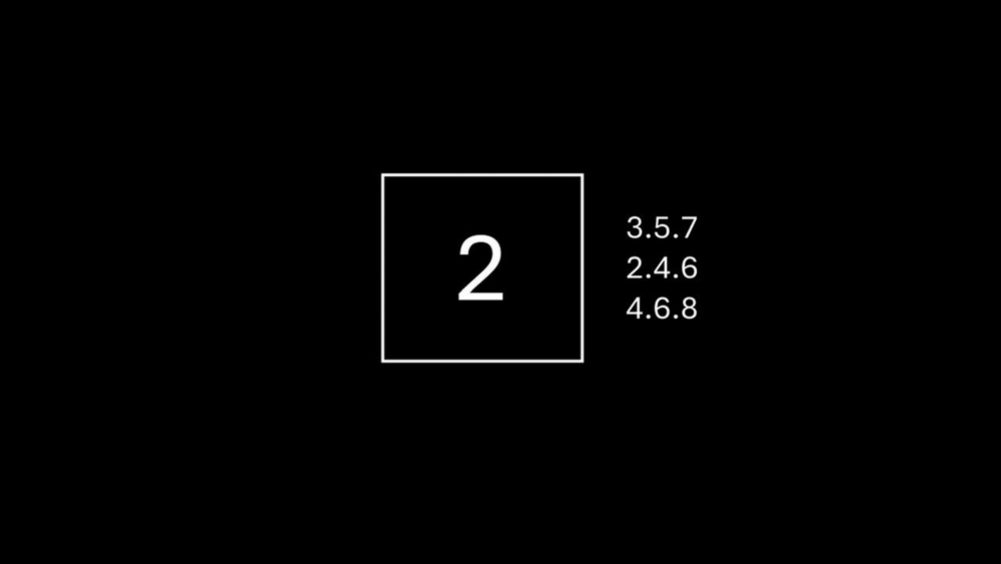
Schematic of the Cambridge Neuropsychological Test Automated Battery Rapid Visual Information Processing task.

The CANTAB RVP is a 10 minute test which measures sustained attention by presenting a rapid stream of digits and requiring participants to detect target sequences. A white box is displayed in the center of the screen in which digits 2 through 9 are rapidly presented at 100 digits per minute. Participants are required to detect target sequences (e.g., 3‐5‐7, 2‐4‐6, or 4‐6‐8) and respond to this target sequence as quickly as possible. A schematic of the CANTAB RVP is presented in Figure 1. The outcome measure of interest is the sustained attention measure, RVP A', which is the signal detection measure of a subject's sensitivity to the target sequence, regardless of response tendency.^19^ In addition, we also examine the RVP median latency, which is the median response latency on trials in which the subject responded correctly.

### 2.3 Behavioral analysis

Linear mixed‐effects models were used to examine both cross‐sectional and longitudinal changes in behavioral performance across groups. Models included fixed effects for time, group (HDGE vs. HC), and the group × time interaction to assess differential trajectories over time. Covariates included age, sex, IQ, and interval between cognitive visits, which were entered as fixed effects. All analyses were conducted using the lmer function from the nlme R package.

Model comparisons and significance testing were performed using the analysis of variance (ANOVA) function to evaluate main effects and interactions. To account for multiple comparisons, the Benjamini–Hochberg procedure was applied, and the false discovery rate (FDR) was controlled a priori at *q *< 0.15,^21^ consistent with previous HD‐YAS publications.^6^, ^7^, ^14^ Both uncorrected and adjusted *p* values are reported, and effect sizes are reported as partial eta squared for interpretability (${ŋ}_{p}^{2})$.

In addition, in the HDGE group only, we assessed the Pearson correlation between sustained attention (RVP A') and age, CAG, CAG‐Age Product (CAP100), age × CAG, and HD‐ISS using cor.test in R, separately for baseline and follow‐up performance.

### 2.4 Image acquisition

All MRI data were acquired on a 3‐Tesla Prisma scanner (Siemens Healthcare) with a radiofrequency body coil for transmission and a 64‐channel head coil for signal reception using a protocol optimized for this cohort.^6^, ^7^ The T1‐weighted (T1w) images were acquired using a 3D magnetization‐prepared rapid gradient echo sequence with a repetition time (TR) = 2530 ms and time to echo (TE) = 3.34 ms; inversion time of 1100 ms, flip angle of 7°, field of view = 256 mm^2^; 64 slices of 1.0 mm thickness were collected. The resting‐state T2*‐weighted images were acquired with a TR = 3360 ms and TE = 30 ms; field of view = 192 mm^2^, flip angle of 90°; 48 slices of 2.5 mm thickness were collected anterior to posterior in the transverse orientation. Field maps were collected to correct for inhomogeneity in the B0 field of the echo planar imaging (EPI) fMRI images: TR = 1020 ms; TE1 = 10 ms; TE2 = 12.46 ms. Sixty‐four slices were acquired with 2 mm slice thickness with an in‐plane field of view of 192 × 192 mm^2^, with 3 × 3 mm^2^ resolution.

### 2.5 Image pre‐processing

Pre‐processing was conducted in SPM 12 (https://www.fil.ion.ucl.ac.uk/spm/software/spm12/). The first five EPI images (out of 165 images) were discarded to allow for steady‐state equilibrium. Functional images were slice‐timing corrected, realigned and unwrapped to correct for head movements and EPI distortions; co‐registered and segmented to normalize images into standard space based on the Montreal Neurological Institute (MNI) template, for group level analysis; and smoothed with an 8 mm full‐width half‐maximum (FWHM) Gaussian kernel, to account for residual inter‐subject differences. The default SPM12 steps were used. We used the FMRIB Software Library (FSL) motion outliers’ function to determine the framewise displacement of each image. We determined that participants with mean framewise displacement (FD) > 0.20 mm would be excluded.^22^ Movement was small in the cohort and no participants were excluded. However, to reduce movement confounds, FD was added as a covariate in the imaging analyses.

We specified 22 regions of interest (ROIs) based on previous imaging studies of the RVP task.^19^, ^20^ These were the left and right middle frontal gyrus, inferior frontal gyrus (IFG; parcellated into orbitalis, triangularis, and operculum), supplementary motor area (SMA), middle occipital gyrus, superior parietal gyrus, inferior parietal gyrus, lentiform nucleus (averaged from the putamen and globus pallidus), caudate and thalamus. ROIs were defined using the automated anatomical labeling (AAL) atlas 3.^23^

After the pre‐processing steps, the data were entered into the CONN toolbox^24^ release 22.v2407^25^ implemented in SPM.^26^ Potential outlier scans were identified using ART^27^ as acquisitions with FD > 0.9 mm or global blood oxygenation level dependent (BOLD) signal changes above five standard deviations,^22^ and a reference BOLD image was computed for each subject by averaging all scans excluding outliers. In addition, functional data were denoised using a standard denoising pipeline,^28^ including the regression of potential confounding effects characterized by white matter timeseries (16 CompCor noise components), cerebrospinal fluid (CSF) timeseries (16 CompCor noise components), realignment regressors (6 components), outlier scans (below 29 factors),^22^ session and task effects (2 factors), and linear trends (2 factors) within each functional run, followed by bandpass frequency filtering of the BOLD timeseries^29^ between 0.008 Hz and 0.1 Hz. CompCor^30^, ^31^ noise components within white matter and CSF were estimated by computing the average BOLD signal as well as the largest principal components orthogonal to the BOLD average, and outlier scans within each subject's eroded segmentation masks. From the number of noise terms included in this denoising strategy, the effective degrees of freedom of the BOLD signal after denoising were estimated to range from 125.5 to 145.9 (average 143.6) across all subjects.

ROI‐to‐ROI connectivity matrices were estimated characterizing the FC between each pair of regions among 24 ROIs. FC strength was represented by Fisher‐transformed bivariate correlation coefficients from a general linear model (weighted‐GLM),^32^ estimated separately for each pair of ROIs, characterizing the association between their BOLD signal timeseries. Individual scans were weighted by a boxcar signal characterizing each individual task or experimental condition, convolved with an SPM canonical hemodynamic response function, and rectified. The putamen and globus pallidus values were then averaged to comprise the lentiform nucleus, which resulted in a 22 x 22 weighted connectivity matrix for each participant. These values from the standardized weighted connectivity matrices were used to perform the correlation analyses with RVP detection threshold in R.

### 2.6 Network analysis

Linear mixed‐effects models were used to examine both cross‐sectional and longitudinal changes in the association between sustained attention and FC across groups. Models included fixed effects for time, group (HDGE vs. HC), and the group × time interaction to assess differential trajectories over time. Covariates included the mean FD for each participant, which were entered as fixed effects. All analyses were conducted using the lmer function from the nlme R package.

Model comparisons and significance testing were performed using the ANOVA function to evaluate main effects and interactions. To account for multiple comparisons, the Benjamini–Hochberg procedure was applied, and the false discovery rate (FDR) was controlled a priori at *q *< 0.15,^21^ consistent with previous HD‐YAS publications.^6^, ^7^, ^14^ Both uncorrected and adjusted *p*‐values are reported for those surviving multiple corrections, and effect sizes are reported as partial eta squared for interpretability (${ŋ}_{p}^{2})$.

### 2.7 Data availability

We are committed to data sharing while maintaining confidentiality due to the sensitive and potentially identifiable nature of these data. Biofluid samples will not be shared due to the limited amount of material available. The remaining samples will be required for replication for the next HD‐YAS visit. Upon reasonable request, data will be made available 24 months after the end of data collection, through application via University College London (UCL) to the principal investigator, Professor Sarah Tabrizi. Researchers will be required to submit a proposal meeting the research criteria and must demonstrate full General Data Protection Regulation compliance. A data access agreement with UCL will be required.

## 3 RESULTS

### 3.1 Behavioral results

#### 3.1.1 Longitudinal mixed model

**FIGURE 2:**
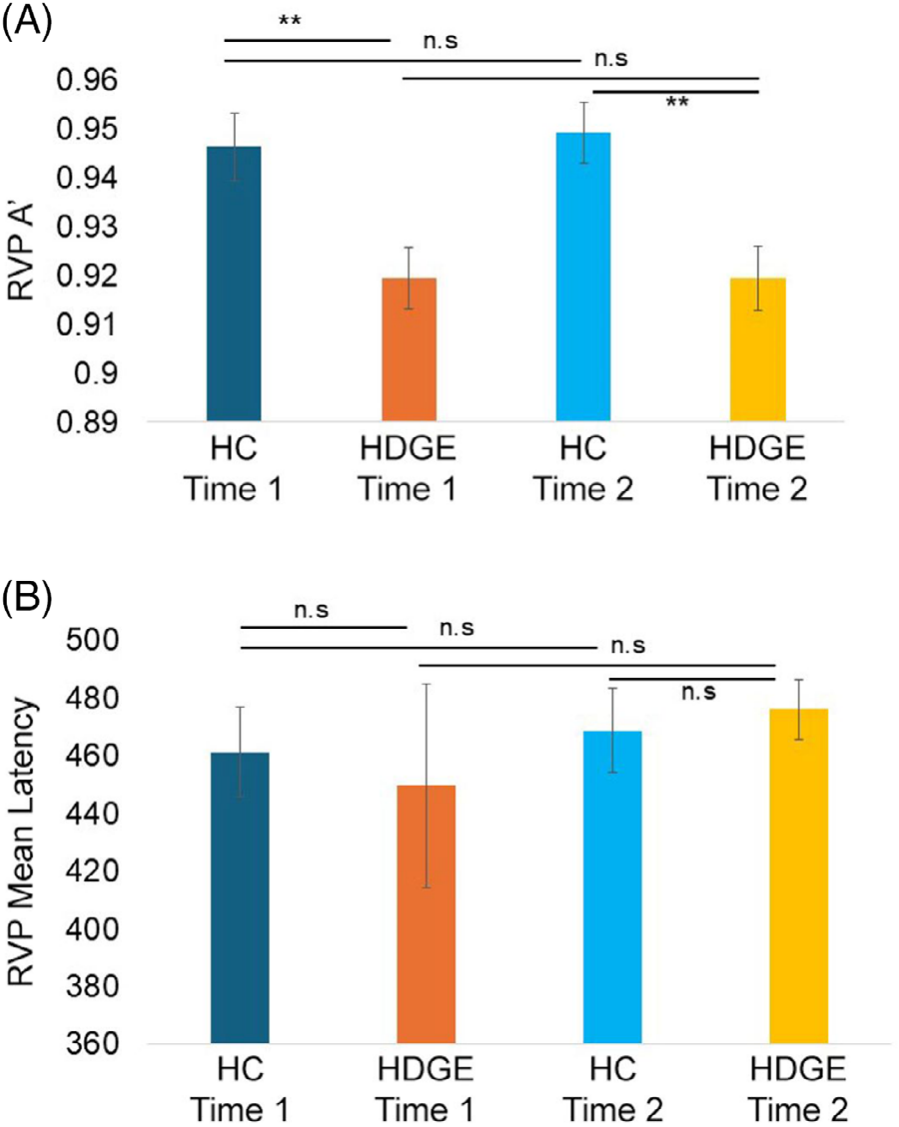
Performance on the Cambridge Neuropsychological Test Automated Battery (CANTAB) Rapid Visual Information Processing (RVP) task: (A) represents the sustained attention performance (RVP A'), (B) represents the reaction time performance (RVP Median Latency). The HDGE Time 1 is displayed in orange, HDGE time 2 in yellow, and HC time 1 in blue and HC time 2 in cyan. * *p *< 0.05, ** *p *< 0.01. HC, healthy control; HDGE, Huntington's disease gene expansion.

There was significantly poorer sustained attention (RVP A') in the HDGE group compared to controls (Figure 2), as evidenced by the significant group effect (*β *= –0.03, *F*[1,65] = 11.46, *p *= 0.001, *p_adj _* = 0.006, ${ŋ}_{p}^{2}$= 0.15, 95% CI [–0.04, –0.008]). There was no significant main effect of time, over the 4.7 year period (*β *= 0.003, *F*[1,69] = 0.11, *p *= 0.75, *p_adj _* = 0.76, ${ŋ}_{p}^{2}$= 0.001, 95% CI [–0.01, 0.02]); similarly, the group × time interaction effect was not statistically significant (*β *= –0.003, *F*[1,69] = 0.10, *p *= 0.76, *p_adj _* = 0.76, ${ŋ}_{p}^{2}$= 0.001, 95% CI [–0.02, 0.02]). Importantly, for the median latency measure there was no main effect of group (*β *= –2.75, *F*[1,65] = 0.21, *p *= 0.65, *p_adj _* = 0.76, ${ŋ}_{p}^{2}$= 0.001, 95% CI [–36.6, 30.54]), time (*β *= 7.49, *F*[1,69] = 3.10, *p *= 0.08, *p_adj _* = 0.24, ${ŋ}_{p}^{2}$= 0.04, 95% CI [–21.83, 36.82]), or interaction effect group × time (*β *= 18.88, *F*[1,69] = 0.96, *p *= 0.33, *p_adj _* = 0.66, ${ŋ}_{p}^{2}$= 0.01, 95% CI [–18.80, 56.57]), suggesting that the poorer performance was unrelated to sensorimotor differences.

#### 3.1.2 Correlations with disease metrics

There were no significant correlations at either time point between sustained attention performance and age (*r_t1 _* = –0.10, *p_t1 _* = 0.54; *r_t2 _* = –0.06, *p_t2 _* = 0.71), CAP100 (*r_t1 _* = 0.10, *p_t1 _* = 0.52; *r_t2 _* = 0.05, *p_t2 _* = 0.75), age × CAG (*r_t1 _* = –0.04, *p_t1 _* = 0.79; *r_t2 _* = –0.03, *p_t2 _* = 0.82), or HD‐ISS (*r_t1 _* = 0.10, *p_t1 _* = 0.49; *r_t2 _* = 0.20, *p_t2 _* = 0.20). There was a trend association between sustained attention and CAG at time 1 (*r_t1 _* = 0.28, *p_t1 _* = 0.07) but not at time 2 (*r_t2 _* = 0.13, *p_t2 _* = 0.40).

### 3.2 Functional connectivity results

#### 3.2.1 Longitudinal mixed model

**FIGURE 3:**
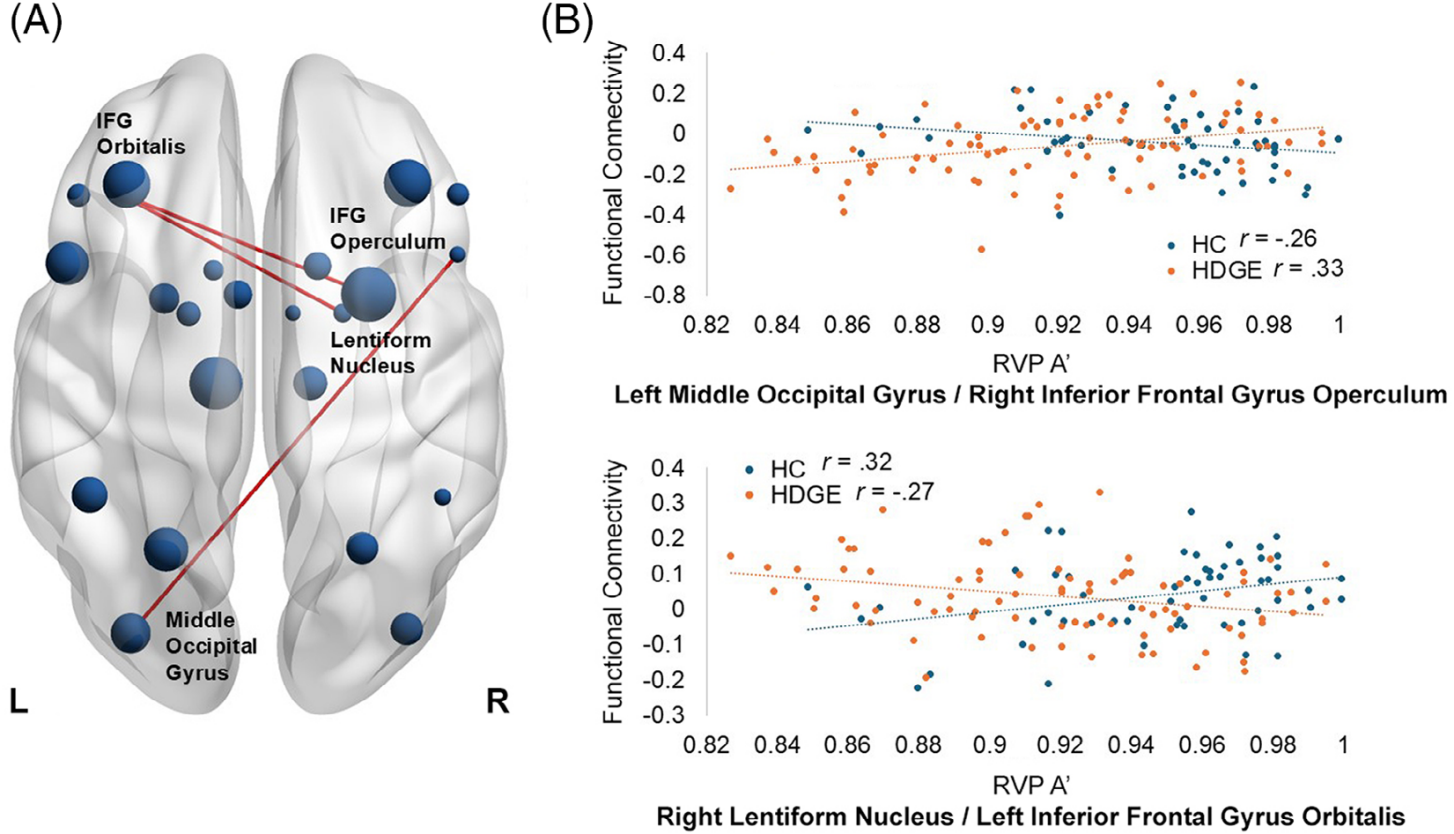
Main effect of group for the association between FC and sustained attention performance: (A) represents the ROIs and the significant connections, (B) represents the strength of the correlation. HDGE is displayed in orange and HC in blue. FC, functional connectivity; HC, healthy control; HDGE, Huntington's disease gene expansion; IFG, inferior frontal gyrus; ROI, region of interest; RVP A', sustained attention detection threshold of the Cambridge Neuropsychological Test Automated Battery Rapid Visual Information Processing.

**FIGURE 4:**
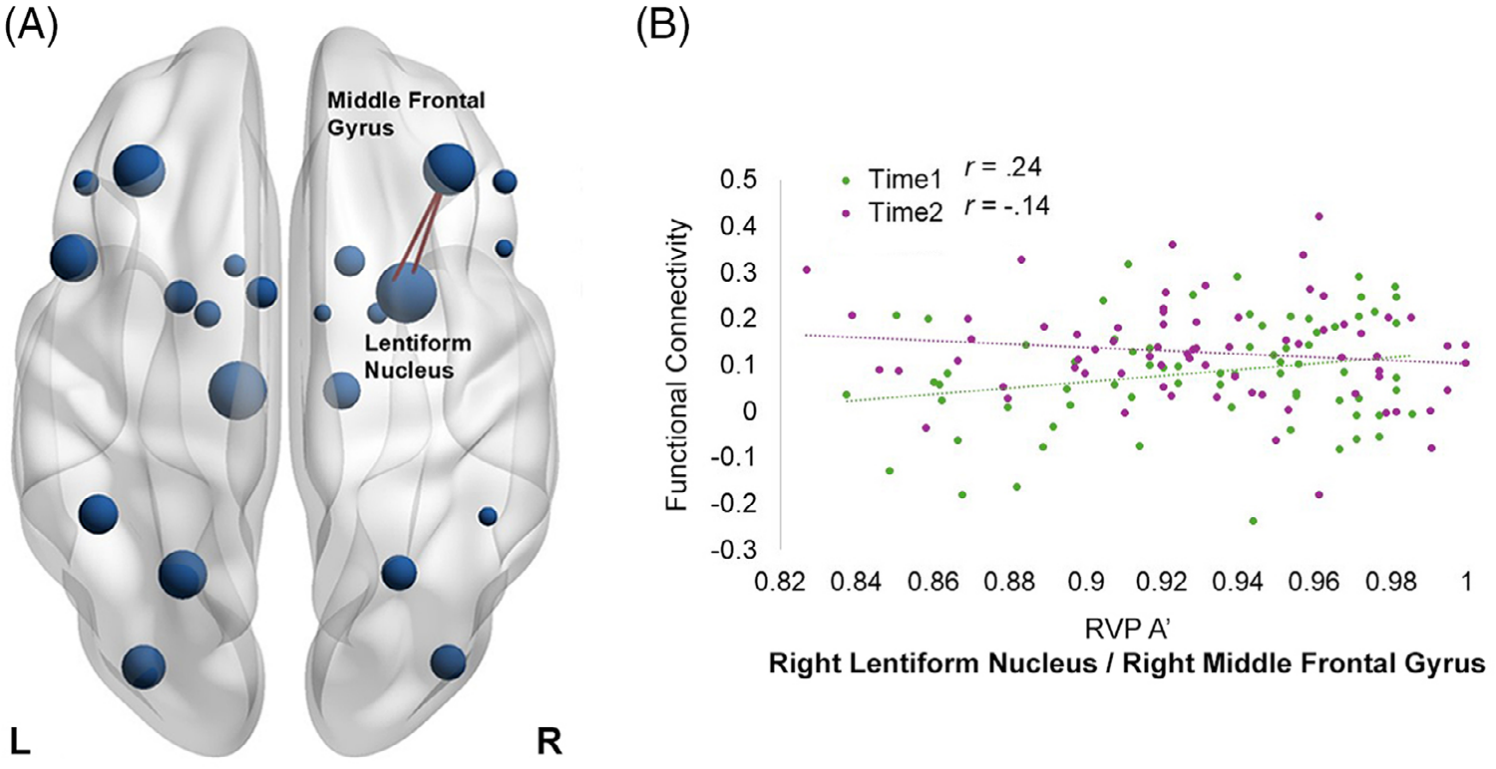
Main effect of time for the association between FC and sustained attention performance: (A) represents the ROIs and the significant connections, (B) represents the strength of the correlation. Time 1 is displayed in green and time 2 in purple. FC, functional connectivity; HDGE, Huntington's disease gene expansion; ROI, region of interest; RVP A', sustained attention detection threshold of the Cambridge Neuropsychological Test Automated Battery Rapid Visual Information Processing.

**FIGURE 5:**
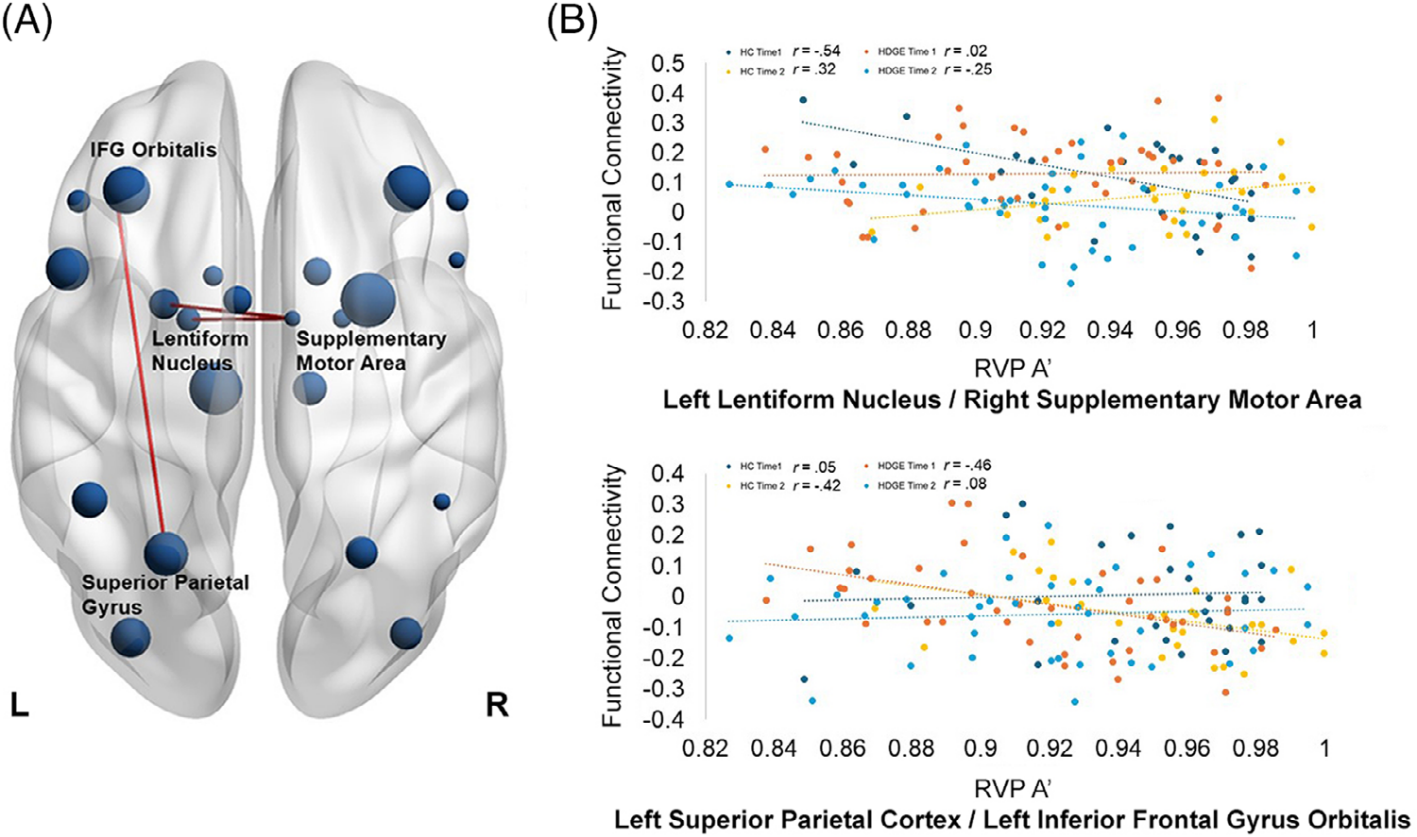
Interaction effect of group × time for the association between FC and sustained attention performance: (A) represents the ROIs and the significant connections, (B) represents the strength of the correlation. HDGE time 1 is displayed in orange, HDGE time 2 in yellow, and HC time 1 in blue, and HC time 2 in cyan. FC, functional connectivity; ROI, region of interest. FC, functional connectivity; HDGE, Huntington's disease gene expansion; IFG, inferior frontal gyrus; ROI, region of interest; RVP A', sustained attention detection threshold of the Cambridge Neuropsychological Test Automated Battery Rapid Visual Information Processing.

For the neuroimaging data, there was a significant main effect of group (Figure 3) for the correlation between sustained attention and FC between the left middle occipital and right operculum (*r*_HC _= –0.26, *r*_HDGE _= 0.33, *β *= 2.52, *F*[1,134] = 11.13, *p *= 0.001, *p_adj _* = 0.099, ${ŋ}_{p}^{2}$= 0.08, 95% CI [0.72, 4.33]) and the right lentiform nucleus and left orbitalis region (*r*_HC _= 0.32, *r*_HDGE _= –0.27, *β *= –1.41, *F*[1,106.51] = 10.54, *p *= 0.002, *p_adj _* = 0.099, ${ŋ}_{p}^{2}$= 0.09, 95% CI [–2.74, –0.09]). There was a significant main effect of time (Figure 4) for the right lentiform nucleus and the right middle frontal gyrus (*r*_T1 _= 0.24, *r*_T2 _= –0.14, *β *= –2.10, *F*[1,134] = 9.58, *p *= 0.002, *p_adj _* = 0.011, ${ŋ}_{p}^{2}$= 0.07, 95% CI [–3.73, –0.47]). Importantly, there were significant interaction effects for group × time (Figure 5) for the left lentiform nucleus and right SMA (*r*_HCT1 _= –0.54*, r*_HCT2 _= 0.32, *r*_HDGET1 _= 0.02*, r*_HDGET2 _= –0.25, *β *= –3.71, *F*[1,134] = 11.35, *p *= 0.0001, *p_adj _* = 0.099, ${ŋ}_{p}^{2}$= 0.08, 95% CI [–5.83, –1.60]) and the left superior parietal cortex and the left orbitalis region (*r*_HCT1 _= 0.05*, r*_HCT2 _= –0.42, *r*_HDGET1 _= –0.46*, r*_HDGET2 _= 0.08, *β *= 3.41, *F*[1,90.69] = 8.97, *p *= 0.004, *p_adj _* = 0.13, ${ŋ}_{p}^{2}$= 0.09, 95% CI [1.23, 5.60]). All statistically significant results, including those that did not survive correction, are included in Table S2 in supporting information.

## 4 DISCUSSION

We examined sustained attention in a large very‐far‐from‐clinical‐motor‐onset HDGE group from HD‐YAS,^6^, ^7^ the earliest adult HDGE cohort studied to date. The key findings were that sustained attention, but not reaction time, was disrupted early in HDGE individuals cross‐sectionally, potentially as early as previous changes observed for cognitive flexibility.^14^, ^15^, ^16^ This poorer performance was associated with aberrant FC in brain regions known to be important in attentional function. In addition to the cross‐sectional changes, there were longitudinal alterations in FC supporting attention in the HDGE group.

### 4.1 Behavioral findings

Our behavioral results demonstrated that the HDGE group had an early deficit in sustained attention compared to controls, as evidenced by the main effect of group, with a large effect size (${ŋ}_{p}^{2}$= 0.15, ≈ *d *= 0.84). This is consistent with our own previous findings in the full YAS‐HD cohort, in which at baseline there was statistically poorer sustained attention in HDGE, albeit that did not survive correction for multiple comparisons.^6^ At 4.5 year follow‐up, the cross‐sectional difference was more pronounced and survived multiple comparison correction.^7^ This was the first evidence of an early deficit in sustained attention in HDGE individuals. Much of the literature has focused on the early impairment in cognitive flexibility,^14^, ^15^, ^16^ given the involvement of the striatum in this cognitive function.^12^, ^13^, ^14^ There have been some reports of impaired sustained attention in HD,^17^, ^18^ at later stages of the disease. Hart et al.^17^ do include a group of “premanifest” patients earlier in disease progression, but do not show any differences in sustained attention compared to controls. This may be due to the sensitivity of the CANTAB RVP to detect very early changes in sustained attention. Despite the cross‐sectional differences, there was no measurable evidence of further longitudinal decline over the 4.7 years in the HDGE group, as evidenced by the non‐significant group × time interaction. Given this stability, it is possible that the disrupted sustained attention may represent a neurodevelopmental deficit rather than a neurodegenerative process in HD. Indeed, impairments in CANTAB RVP are present and are known to be associated with the IFG in the neurodevelopmental disorder of attention deficit hyperactivity disorder,^33^ which has high heritability^34^ and is characterized by symptoms of inattention, hyperactivity, and impulsivity. However, the trend association with CAG in the present study, and indeed our association with age and CAG in the full cohort,^7^ suggests that sustained attention may at least in part be related to HD progression. Further longitudinal follow‐up may be able to better elucidate the relative contribution of neurodevelopmental and neurodegenerative impairments.

### 4.2 Functional connectivity

FC analyses revealed altered neural correlates of sustained attention in HDGE individuals. Specifically, the significant main effect of group demonstrated alterations to the relationship between sustained attention and FC between the left middle occipital and right IFG operculum, and between the right lentiform nucleus and left IFG orbitalis, which suggests disrupted integration between the IFG and occipital and striatal regions. Although this study used resting‐state FC, the interpretation of results is informed by task‐based fMRI literature in healthy individuals, which has consistently implicated these regions in sustained attention.

The left middle occipital gyrus is a core region for visual processing, responsible for early visual input and the perceptual encoding of visual stimuli.^35^ Its connectivity with the right IFG operculum, a region implicated in attention selection, response inhibition, and task switching,^36^ suggests a mechanism by which visual information is modulated for goal‐directed behavior. Disruptions in this pathway may impair the top‐down control of visual attention, reducing the ability to filter irrelevant stimuli or maintain focus over time. In HC, FC between the left middle occipital gyrus and right IFG operculum was negatively correlated with sustained attention (*r* = –0.26), suggesting that greater connectivity between these visual and frontal regions may reflect inefficient engagement of a network not typically required for optimal attention. In other words, better performance in HC may rely on more streamlined or specialized circuits, with reduced reliance on visual–frontal coupling. In contrast, HDGE individuals showed a positive correlation (*r* = 0.33), indicating that they may be recruiting this pathway as a compensatory mechanism to support attention. This increased engagement could reflect an attempt to support attentional control by drawing on additional sensory–executive resources in response to early deficits elsewhere. However, whether this compensatory strategy is effective or sustainable remains uncertain.

The involvement of the right lentiform nucleus, a structure within the basal ganglia associated with motor control, and cognitive resource allocation^37^ and the left IFG orbitalis, a region known for its role in executive function, emotional regulation, and value‐based decision making,^38^, ^39^ suggests alterations in frontostriatal circuitry. These circuits are essential for sustaining attention under cognitive load, dynamically adjusting effort, and managing competing demands. The FC between the right lentiform nucleus and left IFG orbitalis was positively associated with sustained attention in HC (*r* = 0.32), suggesting that stronger frontostriatal coupling supports better sustained attention. This is consistent with known roles of frontostriatal circuits in cognitive control, effort regulation, and the suppression of distractions. However, HDGE individuals showed a negative correlation (*r* = –0.27) in this pathway, implying that greater engagement of this circuit was linked to poorer performance, potentially reflecting a pathological shift in how these regions interact. Rather than facilitating sustained attention, the increased FC in HDGE may represent ineffective or dysregulated recruitment of this network under increased cognitive demand.

In addition, there were significant group × time interactions in the association between sustained attention and FC between parietal, frontal, and motor regions, specifically the left lentiform nucleus and right SMA and the left superior parietal cortex and the left IFG orbitalis. The involvement of the left lentiform nucleus and the right SMA points to possible disruptions in motor–cognitive integration, which is critical for sustaining goal‐directed behavior over time.^40^ The lentiform nucleus, which includes the putamen and globus pallidus, plays a vital role in regulating motor function and attention.^37^ Alterations in its FC, particularly with frontal motor areas like the SMA, could reflect compensatory recruitment or early motor dysfunction, particularly in HD where neurodegeneration affect the frontostriatal circuits.

When considering the specific correlation coefficients between sustained attention and FC and how they change over time, the HC at time 1 shows a moderate‐to‐strong negative correlation (*r* = –0.54), indicating that greater FC between the lentiform nucleus and SMA is associated with poorer sustained attention. This could reflect an early, suboptimal state of network engagement, in which hyperconnectivity between subcortical motor and frontal regions may interfere with attentional efficiency during initial task exposure. Interestingly, by time 2, this correlation shifts to positive (*r* = 0.32), suggesting a refinement of the network, when increased FC may now support rather than hinder attentional performance, consistent with compensatory or adaptive plasticity in HC. This is unlikely to be due to a learning effect as in the CANTAB RVP the digit presentation is randomized, and the task has good test–retest reliability over time.^41^

In contrast, HDGE individuals show no meaningful association at time 1 (*r* = 0.02), suggesting that the lentiform nucleus–SMA network may not be engaged in sustained attention in the same way. By time 2, a negative correlation emerges (*r* = –0.25), potentially indicating a lagged or disrupted developmental trajectory. Rather than moving toward a more efficient or supportive FC–attention relationship, HDGE participants appear to follow a delayed pattern that mirrors the earlier, less optimized state seen in HC. This temporal lag suggests that HDGE individuals may be delayed in the normal trajectory of network optimization, possibly due to early neurobiological disruptions. The fact that they begin to exhibit the same negative correlation HC showed at baseline, but only at a later time point, may reflect a delayed or impaired compensatory process.

The left superior parietal cortex is essential for attentional control,^42^ while the left IFG (orbitalis) is involved in inhibitory control, task switching, and higher‐order executive functions.^36^, ^38^ Altered FC between these regions may indicate a breakdown or reorganization of the dorsal attention network and frontoparietal control network, both of which are heavily implicated in sustained attention. These patterns of correlation coefficients support a pathological interpretation in HDGE individuals, though with a nuanced temporal profile. In HC, the correlation between superior parietal cortex and IFG orbitalis connectivity and sustained attention shifts from near zero at time 1 (*r* = 0.05) to a moderate negative correlation at time 2 (*r* = –0.42), suggesting that as this network becomes more engaged over time, it may do so inefficiently, reflecting compensatory activation under increasing cognitive demands. In contrast, HDGE individuals show a moderate negative correlation at time 1 (*r* = –0.46), indicating early, potentially strained engagement of this frontoparietal circuit. By time 2, however, this association weakens considerably (*r* = 0.08), suggesting a breakdown or disengagement of the network. This trajectory implies that HDGE participants prematurely enter a dysfunctional network state that HCs only develop later and, unlike HCs, fail to reorganize or sustain adaptive engagement. The overall pattern is consistent with early, inefficient compensation followed by functional decline, supporting a pathological trajectory in HDGE individuals.

Overall, the results demonstrate frontostriatal circuit alterations in HDGE individuals, confirming previous publications.^11^, ^14^, ^43^, ^44^ Our findings provide the first evidence that these circuit connectivity changes also disrupt sustained attention. In addition, there are early alterations in frontoparietal and fronto‐occipital networks in HDGE that affect sustained attention.

## 5 CONCLUSION

Our novel findings demonstrate a cross‐sectional, but not longitudinal, deficit in sustained attention in HDGE individuals, which was associated with aberrant FC in brain regions known to be important in attentional function. These findings support known evidence of early cognitive dysfunction in HD, such as deficits in cognitive flexibility. It is possible that the deficits noted in cognitive flexibility may potentially result from the problems in sustained attention.^45^ Furthermore, our results highlight potential compensatory and pathological changes in distributed brain circuits prior to clinical motor diagnosis. In addition, these mechanisms provide novel insights into the cognitive pathophysiology of HD and identify potential early cognitive biomarkers for intervention. This study is the first to show that sustained attention is disrupted early in HDGE individuals.

## CONFLICT OF INTEREST STATEMENT

The authors declare no conflicts of interest. Author disclosures are available in the supporting information.

## Supporting information



Supporting Information

Supporting Information

## References

[1] Paulsen JS , Long JD , Ross CA , et al. Prediction of manifest Huntington's disease with clinical and imaging measures: a prospective observational study. Lancet Neurol. 2014;13:1193‐1201. 25453459 10.1016/S1474-4422(14)70238-8 PMC4373455

[2] Langbehn DR , Hayden MR , Paulsen JS , PHIotHS Group . CAG‐repeat length and the age of onset in Huntington disease (HD): a review and validation study of statistical approaches. Am J Med Genet B Neuropsychiatr Genet. 2010;153:397‐408. 10.1002/ajmg.b.30992 PMC3048807 19548255

[3] Tabrizi SJ , Schobel S , Gantman EC , et al. A biological classification of Huntington's disease: the Integrated Staging System. Lancet Neurol. 2022;21:632‐644. 35716693 10.1016/S1474-4422(22)00120-X

[4] Paulsen JS , Langbehn DR , Stout JC , et al. Detection of Huntington's disease decades before diagnosis: the Predict‐HD study. J Neurol Neurosurg Psychiatry. 2008;79:874‐880. 18096682 10.1136/jnnp.2007.128728 PMC2569211

[5] Ross CA , Aylward EH , Wild EJ , et al. Huntington disease: natural history, biomarkers and prospects for therapeutics. Nat Rev Neurol. 2014;10:204‐216. 24614516 10.1038/nrneurol.2014.24

[6] Scahill RI , Zeun P , Osborne‐Crowley K , et al. Biological and clinical characteristics of gene carriers far from predicted onset in the Huntington's disease Young Adult Study (HD‐YAS): a cross‐sectional analysis. Lancet Neurol. 2020;19:502‐512. 32470422 10.1016/S1474-4422(20)30143-5 PMC7254065

[7] Scahill RI , Farag M , Murphy MJ , et al. Somatic CAG repeat expansion in blood associates with biomarkers of neurodegeneration in Huntington's disease decades before clinical motor diagnosis. Nat Med. 2025;31(3):807‐818. 39825149 10.1038/s41591-024-03424-6 PMC11922752

[8] Tabrizi SJ , Langbehn DR , Leavitt BR , et al. Biological and clinical manifestations of Huntington's disease in the longitudinal TRACK‐HD study: cross‐sectional analysis of baseline data. Lancet Neurol. 2009;8:791‐801. 19646924 10.1016/S1474-4422(09)70170-X PMC3725974

[9] Tabrizi SJ , Scahill RI , Durr A , et al. Biological and clinical changes in premanifest and early stage Huntington's disease in the TRACK‐HD study: the 12‐month longitudinal analysis. Lancet Neurol. 2011;10:31‐42. 21130037 10.1016/S1474-4422(10)70276-3

[10] Blumenstock S , Dudanova I . Cortical and striatal circuits in Huntington's disease. Front Neurosci. 2020;14:82. 32116525 10.3389/fnins.2020.00082 PMC7025546

[11] Ciarochi JA , Calhoun VD , Lourens S , et al. Patterns of co‐occurring gray matter concentration loss across the Huntington disease prodrome. Front Neurol. 2016;7:147. 27708610 10.3389/fneur.2016.00147 PMC5030293

[12] Morris LS , Kundu P , Dowell N , et al. Fronto‐striatal organization: defining functional and microstructural substrates of behavioural flexibility. cortex. 2016;74:118‐133. 26673945 10.1016/j.cortex.2015.11.004 PMC4729321

[13] Vaghi MM , Vértes PE , Kitzbichler MG , et al. Specific frontostriatal circuits for impaired cognitive flexibility and goal‐directed planning in obsessive‐compulsive disorder: evidence from resting‐state functional connectivity. Biol Psychiatry. 2017;81:708‐717. 27769568 10.1016/j.biopsych.2016.08.009 PMC6020061

[14] Langley C , Gregory S , Osborne‐Crowley K , et al. Fronto–striatal circuits for cognitive flexibility in far from onset Huntington's disease: evidence from the Young Adult Study. J Neurol Neurosurg Psychiatry. 2021;92:143‐149. 33130575 10.1136/jnnp-2020-324104 PMC7841479

[15] Lawrence AD , Hodges JR , Rosser AE , et al. Evidence for specific cognitive deficits in preclinical Huntington's disease. Brain. 1998;121:1329‐1341. 9679784 10.1093/brain/121.7.1329

[16] Lawrence AD , Sahakian BJ , Hodges JR , Rosser AE , Lange KW , Robbins TW . Executive and mnemonic functions in early Huntington's disease. Brain. 1996;119:1633‐1645. 8931586 10.1093/brain/119.5.1633

[17] Hart E , Dumas E , Reijntjes R , et al. Deficient sustained attention to response task and P300 characteristics in early Huntington's disease. J Neurol. 2012;259:1191‐1198. 22143614 10.1007/s00415-011-6334-0 PMC3366183

[18] Brohée S , Grimaldi S , Spieser L , et al. Action impulsivity and attention deficits in patients at an early stage of Huntington disease. J Neural Transm. 2025;132:645‐654. 40029427 10.1007/s00702-025-02888-1

[19] Coull JT , Frith C , Frackowiak RSJ , Grasby P . A fronto–parietal network for rapid visual information processing: a PET study of sustained attention and working memory. Neuropsychologia. 1996;34:1085‐1095. 8904746 10.1016/0028-3932(96)00029-2

[20] Neale C , Johnston P , Hughes M , Scholey A . Functional activation during the rapid visual information processing task in a middle aged cohort: an fMRI study. PLoS One. 2015;10:e0138994. 26488289 10.1371/journal.pone.0138994 PMC4619344

[21] Benjamini Y , Hochberg Y . Controlling the false discovery rate: a practical and powerful approach to multiple testing. J R Stat Soc Series B Stat Methodol. 1995;57:289‐300.

[22] Power JD , Mitra A , Laumann TO , Snyder AZ , Schlaggar BL , Petersen SE . Methods to detect, characterize, and remove motion artifact in resting state fMRI. Neuroimage. 2014;84:320‐341. 23994314 10.1016/j.neuroimage.2013.08.048 PMC3849338

[23] Rolls ET , Huang C‐C , Lin C‐P , Feng J , Joliot M . Automated anatomical labelling atlas 3. Neuroimage. 2020;206:116189. 31521825 10.1016/j.neuroimage.2019.116189

[24] Whitfield‐Gabrieli S , Nieto‐Castanon A . Conn: a functional connectivity toolbox for correlated and anticorrelated brain networks. Brain Connectivity. 2012;2:125‐141. 22642651 10.1089/brain.2012.0073

[25] Nieto‐Castanon A , Whitfield‐Gabrieli S . CONN functional connectivity toolbox (RRID: SCR_009550), Version 21; 2021. 10.1089/brain.2012.0073 22642651

[26] Penny WD , Friston KJ , Ashburner JT , Kiebel SJ , Nichols TE . Statistical Parametric Mapping: the Analysis of Functional Brain Images. Elsevier; 2011.

[27] Whitfield‐Gabrieli S , Nieto‐Castanon A , Ghosh S . Artifact detection tools (ART). Cambridge, MA. 2011.

[28] Nieto‐Castanon A . FMRI denoising pipeline. Handbook of Functional Connectivity Magnetic Resonance Imaging Methods in CONN. Hilbert Press; 2020.

[29] Hallquist MN , Hwang K , Luna B . The nuisance of nuisance regression: spectral misspecification in a common approach to resting‐state fMRI preprocessing reintroduces noise and obscures functional connectivity. Neuroimage. 2013;82:208‐225. 23747457 10.1016/j.neuroimage.2013.05.116 PMC3759585

[30] Behzadi Y , Restom K , Liau J , Liu TT . A component based noise correction method (CompCor) for BOLD and perfusion based fMRI. Neuroimage. 2007;37:90‐101. 17560126 10.1016/j.neuroimage.2007.04.042 PMC2214855

[31] Chai XJ , Castañón AN , Öngür D , Whitfield‐Gabrieli S . Anticorrelations in resting state networks without global signal regression. Neuroimage. 2012;59:1420‐1428. 21889994 10.1016/j.neuroimage.2011.08.048 PMC3230748

[32] Nieto‐Castanon A . Functional connectivity measures. Handbook of Functional Connectivity Magnetic Resonance Imaging Methods in CONN. Hilbert Press; 2020.

[33] Pironti VA , Lai M‐C , Müller U , et al. Neuroanatomical abnormalities and cognitive impairments are shared by adults with attention‐deficit/hyperactivity disorder and their unaffected first‐degree relatives. Biol Psychiatry. 2014;76:639‐647. 24199662 10.1016/j.biopsych.2013.09.025 PMC4183379

[34] Faraone SV , Larsson H . Genetics of attention deficit hyperactivity disorder. Mol Psychiatry. 2019;24:562‐575. 29892054 10.1038/s41380-018-0070-0 PMC6477889

[35] Rehman A , Al Khalili Y . Neuroanatomy, Occipital Lobe. In: StatPearls. StatPearls Publishing, Treasure Island (FL); 2025. 31335040

[36] Hampshire A , Chamberlain SR , Monti MM , Duncan J , Owen AM . The role of the right inferior frontal gyrus: inhibition and attentional control. Neuroimage. 2010;50:1313‐1319. 20056157 10.1016/j.neuroimage.2009.12.109 PMC2845804

[37] Young CB , Reddy V , Sonne J . Neuroanatomy, basal ganglia. StatPearls [Internet]: StatPearls Publishing; 2023. 30725826

[38] Diveica V , Riedel MC , Salo T , Laird AR , Jackson RL , Binney RJ . Graded functional organization in the left inferior frontal gyrus: evidence from task‐free and task‐based functional connectivity. Cereb Cortex. 2023;33:11384‐11399. 37833772 10.1093/cercor/bhad373 PMC10690868

[39] Christopoulos GI , Tobler PN , Bossaerts P , Dolan RJ , Schultz W . Neural correlates of value, risk, and risk aversion contributing to decision making under risk. J Neurosci. 2009;29:12574‐12583. 19812332 10.1523/JNEUROSCI.2614-09.2009 PMC2794196

[40] Leisman G , Moustafa AA , Shafir T . Thinking, walking, talking: integratory motor and cognitive brain function. Frontiers in Public Health. 2016;4:179575. 10.3389/fpubh.2016.00094 PMC4879139 27252937

[41] Karlsen RH , Karr JE , Saksvik SB , et al. Examining 3‐month test‐retest reliability and reliable change using the Cambridge Neuropsychological Test Automated Battery. Appl Neuropsychol. 2022;29:146‐154. 10.1080/23279095.2020.1722126 32083946

[42] Shomstein S . Cognitive functions of the posterior parietal cortex: top‐down and bottom‐up attentional control. Front Integr Neurosci. 2012;6:38. 22783174 10.3389/fnint.2012.00038 PMC3389368

[43] Kronenbuerger M , Hua J , Bang JY , et al. Differential changes in functional connectivity of striatum‐prefrontal and striatum‐motor circuits in premanifest Huntington's disease. Neurodegenerative Diseases. 2019;19:78‐87. 31412344 10.1159/000501616

[44] Wolf RC , Sambataro F , Vasic N , Schönfeldt‐Lecuona C , Ecker D , Landwehrmeyer B . Altered frontostriatal coupling in pre‐manifest Huntington's disease: effects of increasing cognitive load. Eur J Neurol. 2008;15:1180‐1190. 18754766 10.1111/j.1468-1331.2008.02253.x

[45] Benitez VL , Vales C , Hanania R , Smith LB . Sustained selective attention predicts flexible switching in preschoolers. J Exp Child Psychol. 2017;156:29‐42. 28024178 10.1016/j.jecp.2016.11.004 PMC5253114

