# Supplementary material for: Discovery of disrupted sustained attention and altered functional connectivity in far‐from‐onset Huntington's disease gene‐expanded young adults
Source: Alzheimers Dement. 2026 Jan 13;22(1):e70944. doi: 10.1002/alz.70944 (PMC12797252; doi:10.1002/alz.70944)
Supplement: Supplementary file 2 — Supporting Information [file ALZ-22-e70944-s002.docx]

**Supplementary Material: Discovery of disrupted sustained attention and altered functional connectivity in far-from-onset Huntington’s disease gene-expanded young adults.**

# Christelle Langley^1,2^, Michela Leocadi^3^, Nicola Z. Hobbs^3^, Mena Farag^3^, Michael J. Murphy^3^, Kate Fayer^3^, Rachael I. Scahill^3^, James B. Rowe^4,5,6^, Trevor W. Robbins^7^, Sarah J. Tabrizi^3^, and Barbara J. Sahakian^1,2^

**Author affiliations:**

1. Department of Psychiatry, University of Cambridge, Cambridge, UK.
2. Behavioural and Clinical Neuroscience Institute, University of Cambridge, Cambridge, UK.
3. Huntington’s Disease Centre, Department of Neurodegenerative Disease, UCL Queen Square Institute of Neurology, University College London, London, UK.
4. Department of Clinical Neurosciences, University of Cambridge, Cambridge Biomedical Campus, Cambridge, UK.
5. Cambridge University Hospitals NHS Foundation Trust, Cambridge, UK.
6. Medical Research Council Cognition and Brain Sciences Unit, University of Cambridge, Cambridge, UK,
7. Department of Psychology, University of Cambridge, Cambridge

## Behavioural Analysis

### Longitudinal mixed model

We included an additional model of fixed effects for time, group (HDGE vs. HC), and the group×time interaction to assess differential trajectories over time, including age, sex, IQ, interval between cognitive visits and education as covariates. The addition of education in the model did not affect the results. For the sustained attention (RVP A’) there was a significant group effect (*β*=–.03, *F*(1,64.65)=11.32, *p*=.001, *p_adj_*=.006, $ŋ_{p}^{2}$= .15, 95% CI [–.05, –.009]), no significant main effect of time (*β*=.004, *F*(1,79.38)=.21, *p*=.65, *p_adj_*=.79, $ŋ_{p}^{2}$= .001, 95% CI [–.01, .02]) and no significant group×time interaction effect (*β*=–.003, *F*(1,69.36)=.07, *p*=.79, *p_adj_*=.79, $ŋ_{p}^{2}$=.001, 95% CI [–.02, .02]). For the median latency measure there was no main effect of group (*β*=–2.41, *F*(1,64.40)=0.20, *p*=.66, *p_adj_*=.79, $ŋ_{p}^{2}$=.001, 95% CI [–35.67, 30.83]), time (*β*=5.41, *F*(1,78.46=2.00, *p*=.16, *p_adj_*=.48, $ŋ_{p}^{2}$=.02, 95% CI [–24.06, 35.05]), or interaction effect group×time (*β*=17.75, *F*(1,69,22)=.85, *p*=.36, *p_adj_*=.72, $ŋ_{p}^{2}$=.01, 95% CI [–19.80, 55.38]).

**Supplementary Table 1. Comparison between full longitudinal YAS-HD Cohort and rsfMRI sample**

|  | **HDGE (n=43)** | **Full HDGE (n=56)** | ***t-value/x2*** | ***p-value*** | **HC (n=28)** | **Full HC (n=47)** | ***t-value/x2*** | ***p-value*** |
| --- | --- | --- | --- | --- | --- | --- | --- | --- |
| **Age (Time 1)** | 30.13 (5.67) | 29.43 (5.39) | .62 | .54 | 29.81 (5.95) | 29.47 (5.79) | .24 | .81 |
| **Age (Time 2)** | 34.80 (5.61) | 34.62 (5.37) | .16 | .88 | 34.62 (6.11) | 34.78 (5.91) | -.11 | .92 |
| **IQ (NART)** | 102.86 (6.63) | 103.10 (8.12) | -.23 | .82 | 105.93 (8.38) | 103.34 (7.45) | 1.92 | .06 |
| **Education (Time 1)** | 4.23 (.95) | 4.25 (.98) | -.09 | .93 | 4.43 (.92) | 4.27 (.05) | .68 | .50 |
| **Education (Time 2)** | 4.67 (.64) | 4.68 (.69) | -.03 | .98 | 4.71 (.85) | 4.64 (.76) | .39 | .70 |
| **Sex** | 48.84% Female (21) | 48.21% Female (27) | .02 | .89 | 57.14% Female (16) | 59.14% Female (28) | .01 | .97 |
| **Interval (years)** | 4.70 (.54) | 4.68 (.57) | .15 | .88 | 4.84 (.63) | 4.78 (.63) | .51 | .61 |
| **RVP A’ (Time 1)** | .92 (.04) | .92 (.04) | -.30 | .77 | .95 (.04) | .94 (.04) | -.01 | .61 |
| **RVP A’ (Time 2)** | .92 (.04) | .92 (.04) | -.21 | .84 | .95 (.03) | .94 (.04) | -.01 | .45 |
| **RVP RT (Time 1)** | 449.61 (73.71) | 441.85 (68.38) | .54 | .59 | 461.28 (83.09) | 451.84 (80.37) | .48 | .63 |
| **RVP RT (Time 2)** | 475.99 (67.49) | 462.87 (66.02) | .97 | .34 | 468.77 (77.38) | 458.88 (72.27) | .55 | .59 |
| **CAG** | 42.05 (1.54) 39-46 | 42.08 (1.56) | -.19 | .85 |  |  |  |  |
| **CAP100 (Time 1)** | 55.72 (8.26) 41.57-76.90 | 55.65 (8.06) 41.57-76.90 | .04 | .97 |  |  |  |  |
| **CAP100 (Time 2)** | 64.55 (8.25) 48.55-86.70 | 64.51 (8.19) 48.55-86.70 | .02 | .98 |  |  |  |  |
| **HD-ISS Stage 0 (Time 1)** | 86.05% (37) | 83.02% (44); n=53 |  |  |  |  |  |  |
| **HD-ISS Stage 1 (Time 1)** | 13.95% (6) | 15.09% (8); n=53 |  |  |  |  |  |  |
| **HD-ISS Stage 2 (Time 1)** | 0% (0) | .02% (1); n=53 | .86 | .65 |  |  |  |  |
| **HD-ISS Stage 0 (Time 2)** | 65.12% (28) | 64.15% (34); n=53 |  |  |  |  |  |  |
| **HD-ISS Stage 1 (Time 2)** | 34.88% (15) | 33.96% (18); n=53 |  |  |  |  |  |  |
| **HD-ISS Stage 2 (Time 2)** | 0% (0) | .02% (1); n=53 | .82 | .66 |  |  |  |  |

Note: HDGE – Huntington’s Disease Gene Expansion; HC – Healthy Controls; Full – represents the full longitudinal YAS-HD cohort[1]; Age – represented as Mean (standard deviation); IQ (NART) – Intelligence Quotient assessed using the National Adult Reading Test represented as Mean (standard deviation); Education – International Standard Classification of Education represented as Mean (standard deviation); Sex – Percentage (and count) of female participants in each group; Interval (years) – Average time interval between Time 1 and Time 2 cognitive assessments (in years) represented as Mean (standard deviation); RVP A’ – is the target detection measure on the CANTAB RVP Task represented as Mean (standard deviation); RVP RT – is the median reaction time measure on the CANTAB RVP Task represented as Mean (standard deviation); CAG – Number of cytosine-adenine-guanine (CAG) repeats represented as Mean (standard deviation) minimum-maximum; CAP100 – CAG-Age Product scaled to 100 an index combining CAG repeat length and age to estimate disease burden represented as Mean (standard deviation) minimum-maximum; HD-ISS – Huntington’s Disease Integrated Staging System stage represented as percentage (and count); Time 1 / Time 2 indicate the two assessment points in the longitudinal study. Please note ISS staging was missing for three of the full longitudinal sample.


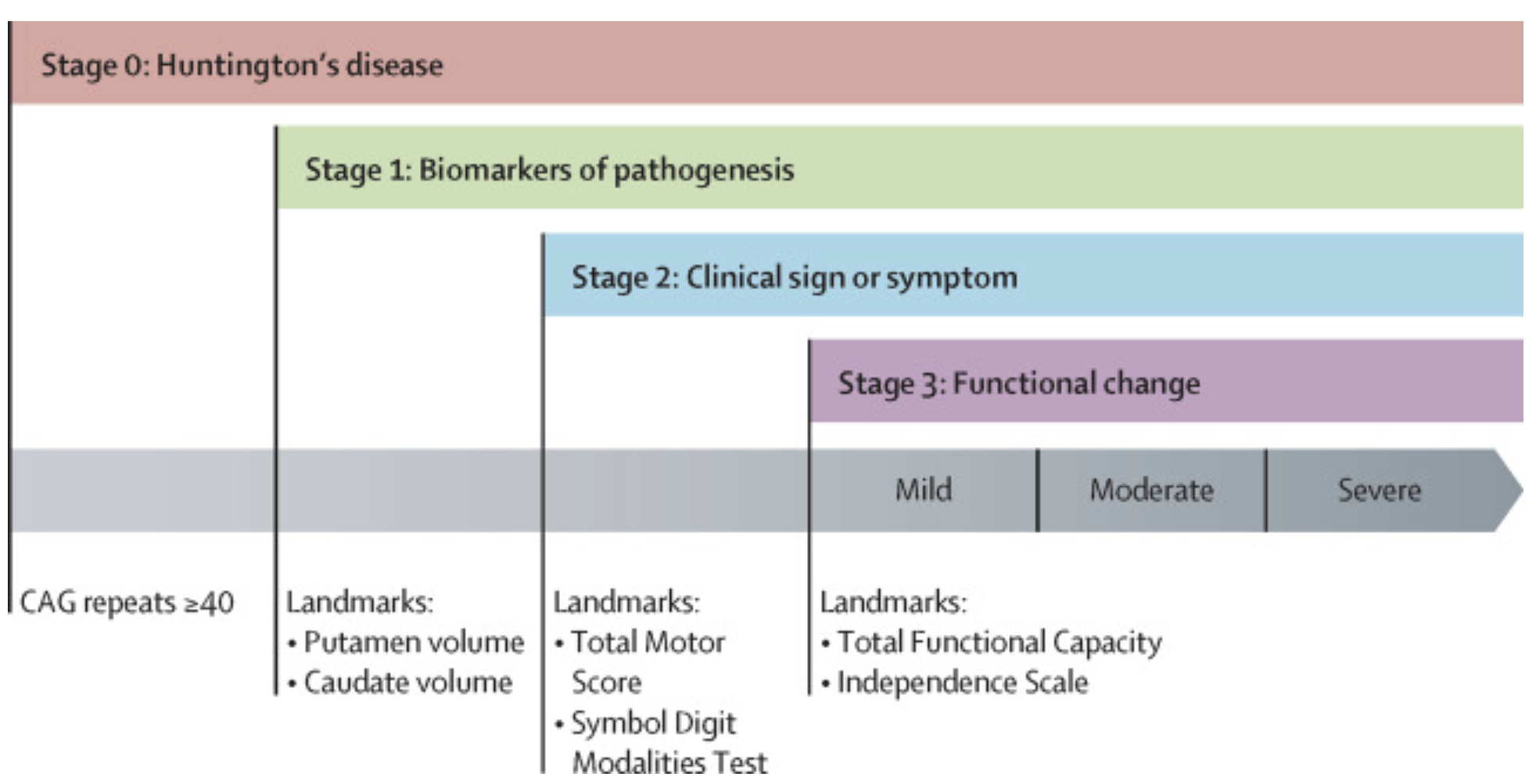


**Supplementary Figure 1. HDD ISS Staging System. Figure from Tabrizi et al., 2022[2].**

**Supplementary Table 2. All significant longitudinal mixed model results for functional connectivity and sustained attention correlations.**

| **Effect** | **FC** | ***β*** | ***df*** | ***F*** | ***p*** | ***p_adj_*** | $\boldsymbol{ŋ}_{\boldsymbol{p}}^{\boldsymbol{2}}$ | **Lower CI** | **Upper CI** |
| --- | --- | --- | --- | --- | --- | --- | --- | --- | --- |
| Group×Time | Left Lentiform Nucleus/Right Supplementary Motor Area | –3.71 | 134.00 | 11.35 | .001 | .10 | .08 | –5.83 | –1.60 |
| Group | Left Middle Occipital Gyrus/Right Operculum | 2.52 | 134.00 | 11.13 | .001 | .10 | .08 | .72 | 4.33 |
| Group | Right Lentiform Nucleus/Left Orbitalis | –1.41 | 106.51 | 10.54 | .002 | .10 | .09 | –2.74 | –.09 |
| Time | Right Lentiform Nucleus/Right Middle Frontal Gyrus | –2.10 | 134.00 | 9.58 | .002 | .11 | .07 | –3.73 | –.47 |
| Group×Time | Left Superior Parietal Cortex/Left Orbitalis | 3.41 | 90.69 | 8.97 | .004 | .13 | .09 | 1.23 | 5.60 |
| Time | Left Inferior Parietal Cortex/Left Middle Occipital Gyrus | 3.37 | 134.00 | 7.45 | .007 | .22 | .05 | .73 | 6.00 |
| Time | Right Operculum/Right Middle Frontal Gyrus | 3.94 | 93.84 | 7.29 | .008 | .22 | .07 | .95 | 6.90 |
| Group | Right Lentiform Nucleus/Left Superior Parietal Cortex | 1.28 | 108.06 | 6.62 | .011 | .23 | .06 | -.06 | 2.62 |
| Group | Left Orbitalis/Right Triangularis | –2.86 | 114.32 | 6.49 | .012 | .23 | .05 | .66 | 4.77 |
| Group | Right Fusiform Gyrus/Right Operculum | 2.55 | 116.36 | 6.47 | .012 | .23 | .05 | .56 | 4.54 |
| Time | Left Middle Occipital Gyrus/Right Supplementary Motor Area | –1.21 | 96.03 | 5.86 | .017 | .25 | .06 | –3.16 | .75 |
| Group | Left Lentiform Nucleus/Right Superior Parietal Cortex | .80 | 112.42 | 5.82 | .018 | .25 | .05 | –.53 | 2.14 |
| Group | Left Middle Occipital Gyrus/Left Middle Frontal Gyrus | –1.87 | 134.00 | 5.46 | .021 | .25 | .04 | –3.93 | .20 |
| Time | Right Lentiform Nucleus/Right Operculum | –1.66 | 104.17 | 5.42 | .022 | .25 | .05 | –3.40 | .07 |
| Group×Time | Left Lentiform Nucleus/Left Middle Frontal Gyrus | –2.52 | 101.02 | 5.40 | .022 | .25 | .05 | –4.61 | –.44 |
| Group | Right Lentiform Nucleus/Left Triangularis | –1.08 | 113.34 | 5.27 | .023 | .25 | .04 | –2.41 | .24 |
| Time | Right Superior Parietal Cortex/Left Middle Frontal Gyrus | 1.75 | 97.29 | 5.21 | .025 | .25 | .05 | –.73 | 4.23 |
| Group | Left Fusiform Gyrus/Right Operculum | 1.46 | 114.28 | 5.02 | .027 | .25 | .04 | –.33 | 3.25 |
| Group | Right Middle Occipital Gyrus/Right Operculum | 2.84 | 105.53 | 4.89 | .029 | .25 | .04 | .82 | 4.85 |
| Time | Right Supplementary Motor Area/Right Triangularis | –1.60 | 96.22 | 4.87 | .030 | .25 | .05 | –3.96 | .71 |
| Time | Right Supplementary Motor Area/Right Middle Frontal Gyrus | –1.85 | 101.19 | 4.86 | .030 | .25 | .05 | –4.30 | .56 |
| Group×Time | Right Supplementary Motor Area/ Left Supplementary Motor Area | –4.34 | 102.23 | 4.84 | .030 | .25 | .05 | –8.13 | –.54 |
| Time | Right Inferior Parietal Cortex/Right Supplementary Motor Area | –2.04 | 100.95 | 4.76 | .031 | .25 | .05 | –4.56 | .43 |
| Group | Left Lentiform Nucleus/Left Supplementary Motor Area | 1.20 | 134.00 | 4.71 | .032 | .25 | .03 | –.31 | 2.71 |
| Group×Time | Left Operculum/Right Middle Frontal Gyrus | –3.60 | 134.00 | 4.64 | .033 | .25 | .03 | –6.80 | –.40 |
| Group | Right Fusiform Gyrus/Right Orbitalis | 2.03 | 134.00 | 4.59 | .034 | .25 | .03 | .14 | 3.92 |
| Group | Right Lentiform Nucleus/Right Superior Parietal Cortex | 1.36 | 105.14 | 4.48 | .037 | .25 | .04 | .05 | 2.68 |
| Group | Left Orbitalis/Left Triangularis | –1.69 | 116.82 | 4.45 | .037 | .25 | .04 | –4.07 | .69 |
| Group×Time | Right Lentiform Nucleus/Right Supplementary Motor Area | –2.15 | 94.20 | 4.34 | .040 | .26 | .04 | –4.16 | –.17 |
| Group | Left Lentiform Nucleus/Left Superior Parietal Cortex | .74 | 112.87 | 4.24 | .042 | .27 | .04 | –.63 | 2.10 |
| Group | Left Lentiform Nucleus/Right Triangularis | –.65 | 104.40 | 4.16 | .044 | .27 | .04 | –2.00 | .71 |
| Time | Right Middle Occipital Gyrus/Left Orbitalis | .90 | 88.57 | 4.07 | .047 | .27 | .04 | –3.53 | 1.65 |
| Time | Left Lentiform Nucleus/Right Supplementary Motor Area | 2.95 | 134.00 | 3.96 | .049 | .27 | .03 | 1.20 | 4.71 |
| Group | Left Lentiform Nucleus/Left Inferior Parietal Cortex | .63 | 105.82 | 3.97 | .049 | .27 | .04 | –.72 | 1.99 |

**References:**

[1] Scahill RI, Farag M, Murphy MJ, Hobbs NZ, Leocadi M, Langley C, et al. Somatic CAG repeat expansion in blood associates with biomarkers of neurodegeneration in Huntington’s disease decades before clinical motor diagnosis. Nature Medicine. 2025:1-12.

[2] Tabrizi SJ, Schobel S, Gantman EC, Mansbach A, Borowsky B, Konstantinova P, et al. A biological classification of Huntington's disease: the Integrated Staging System. The Lancet Neurology. 2022;21:632-44.
